# CBCT vs panoramic radiography in assessment of impacted upper canine and root resorption of the adjacent teeth: A systematic review and meta-analysis

**DOI:** 10.4317/jced.61285

**Published:** 2024-02-01

**Authors:** Mariela Peralta-Mamani, Cássia-Maria-Fischer Rubira, José López-López, Heitor-Marques Honório, Izabel-Regina-Fischer Rubira-Bullen

**Affiliations:** 1 Department of Surgery, Stomatology, Pathology and Radiology - Bauru School of Dentistry, University of São Paulo. Alameda Octávio Pinheiro Brisola, 9-75, Vila Universitária, ZIP CODE: 17012-901, Bauru- SP, Brazil; 2 Oral Health and Masticatory System Group-IDIBELL / Faculty of Medicine and Health Sciences (School of Dentistry) / Odontological Hospital University of Barcelona, University of Barcelona, Campus Bellvitge, Carrer de la Feixa Llarga, s/n, 08907 L’Hospitalet de Llobregat, Barcelona, Spain; 3 Department of Pediatric Dentistry, Orthodontics and Public Health, Bauru School of Dentistry, University of São Paulo. Alameda Octávio Pinheiro Brisola, 9-75, Vila Universitária, ZIP CODE: 17012-901, Bauru- SP, Brazil

## Abstract

**Background:**

The IC may cause reabsorption of adjacent teeth; therefore detailed assessment of its position would enhance decision-making in the clinical workflow. The objective was to compare cone-beam computed tomography (CBCT) and panoramic radiography (PR) in assessing the position of the impacted upper canine (IC) and root resorption of adjacent teeth.

**Material and Methods:**

Pubmed, EMBASE, Science Direct, Web of Science, and SCOPUS databases were searched for studies published before August 2023. Studies that evaluated IC by using both imaging methods were included. For statistical analysis, the Comprehensive Meta-Analysis software (Biostat; Englewood, NJ) was used, p≤0.05.

**Results:**

A total of 17 articles were included, with 877 patients (average age of 17.6 years) and 1,115 ICs. The most frequent mesio-distal location of the IC was in sectors 3 and 4. The meta-analysis was performed with eleven studies. CBCT was more accurate in determining the labio-palatal position compared with PR (*p*<0.001) (CI 95%; 60% in labial position, 0.254-0.542, OR:0.398; 56% in palatal position, 0.350-0.533, OR:0.441; 78% in mid-alveolus position, 0.188-0.234, OR:0.221). For IC angulation to the midline, CBCT showing a smaller and more accurate angle than PR (*p*<0.001) (CI 95%, 18.008-33.686). IC angulation to the occlusal plane and lateral incisor, there was smaller angle in PR compared to CBCT (*p*<0.001) (CI 95%, 51.292-65.934; CI 95%, 30.011-55.954). With PR, fewer cases of root resorption of teeth adjacent to the IC were visualized compared with CBCT (86% less) (*p*<0.001) (CI 95%, 0.089-0.186; OR value: 0.138; n=1049).

**Conclusions:**

CBCT showed statistically significant differences compared to PR in the assessment of IC position and root resorption of adjacent teeth. CBCT provided clinically relevant information that may contribute to diagnosing and planning IC treatment when PR was not sufficient.

** Key words:**Canine teeth, tooth, impacted, panoramic radiography, Cone-beam computed tomography, systematic review, meta-analysis.

## Introduction

The upper canine is the tooth most frequently retained in the maxilla after the third molar and is followed in frequency by the second premolars and central incisors (1,2). In the etiology of impacted canines (IC), multiple factors are considered responsible, among them genetic factors that play a significant role both locally and systemically. The canine will not break out correctly if it deviates from its normal eruption path. This can be caused by a lack of space for tooth eruption or the absence of the lateral incisor. The latter cause is explained by orientation theory, which proposes that the lateral incisor serves as a guide for canine eruption. Other local factors that play a critical role in IC include discrepancies between arch length and tooth size, failed root resorption in the deciduous canine, early loss of the deciduous canine or permanent lateral incisor, dilaceration of the root, and variation in the time of permanent lateral incisor root formation (3-6).

Complications in patients with impacted upper canines include external root resorption in adjacent teeth due to their ectopic position, ankylosis of the affected tooth, and formation of cystic lesions. Because of these complications, early diagnosis of IC and its effects on adjacent structures is essential (7-9).

There are several options for diagnostic imaging of IC, including panoramic radiography (PR) and Cone-beam computed tomography (CBCT). PR images correspond to a two-dimensional aspect of a three-dimensional structure and, hence, have the potential to lead to errors of interpretation of IC due to image distortion and overlap of anatomic structures, factors that are the major limitations of this exam (10). Whereas CBCT allows 3D images to be reformatted without distortions. These characteristics have led to an increase in requests for CBCT. However, dentists should consider the costs and benefits of CBCT before exposing patients to ionizing radiation (11).

To the best of our knowledge, there are no recent systematic reviews that have compared PR and CBCT and reported summarized data on the position of the impacted canine and rate of resorption of adjacent teeth found in the two exams. This information is especially important to clinicians before clinical decision-making in cases of patients with IC.

In the case of IC, CBCT can lead to changing the treatment plan initially decided, based on conventional radiographs (4). This is because CBCT provides more detailed 3D images that include visualization of the resorption of adjacent teeth, of which the lateral incisor is the most commonly affected tooth (9,12,13). Therefore, the study was motivated to provide information obtained from imaging exams commonly used in dentistry that would help clinicians to reach an adequate diagnosis and perform treatment of the impacted upper canine, by establishing its precise location in relation to the adjacent structures. Thus, the aim of this study was to compare CBCT with PR used for the purpose of assessing the position of the impacted upper canine and resorption of adjacent teeth. Therefore, the null hypothesis of this study was that in patients with IC, there is no statistically significant difference in the assessment of its position and resorption of adjacent teeth through PR and CBCT.

## Material and Methods

-Protocol and Registration

This systematic review was conducted according to the Preferred Reporting Items of Systematic reviews and Meta-Analyzes (PRISMA) guidelines (14). Registration was made with PROSPERO, an international database of systematic reviews registered in the area of health and social assistance and developed and managed by the National Institute for Health Research (NIHR) at York University, United Kingdom. The registration number obtained for this systematic review was CRD42016051645 and is available in full on the PROSPERO website: www.crd.york.ac.uk/PROSPERO/.

-Eligibility criteria

Inclusion criteria

The studies selected met the criteria established by the PECO strategy: Participants: patients with IC; Exposure: CBCT; Control: PR; and Outcome: assessment of position of the impacted upper canine and resorption of adjacent teeth. Thus, the search question of this study was: in patients with retained upper canines, is there a difference in CBCT compared with PR for assessing the position of the impacted upper canine and resorption of adjacent teeth? 

All cross-sectional studies that assessed the position of ICs and resorption of adjacent teeth using PR and CBCT were included. Any parameter for evaluating the position was considered (labio-palatal position, mesio-distal position, vertical position, angulation with respect to the lateral incisor, midline or occlusal plane). Studies with ICs in any position, both labial and/or palatal or mid-alveolus, were included. All studies included participants with impacted maxillary canines (unerupted teeth within the maxillary bone), unilateral or bilateral, with or without the presence of the predecessor canine.

Exclusion criteria

Review articles, clinical cases, or case series were excluded. Studies were excluded if their sample was of lower canines or other unerupted teeth, if they evaluated the upper canine only with CBCT or PR but not both, or if the planning of orthodontic treatment was based on questionnaires, and studies that were not from living humans (typodont and skulls). Studies whose participants had cysts or tumors around impacted canines, a history of dental trauma, ectopic canines, previous orthodontic treatment, evaluation after orthodontic treatment, syndromes, and craniofacial anomalies were excluded.

-Exposure and Control

PR images were used as a control as they allow 2D visualization of anatomical structures and present the least risk to patients. CBCT images were considered the exposure/test condition due to their more detailed 3D assessment of the impacted canine position in the maxilla and the increased risk to patients through ionizing radiation exposure.

-Information sources and Search

The identification of the included studies was based on a search strategy for each electronic database: PubMed, EMBASE, Web of Science, SCOPUS, LIVIVO, and Virtual Health Library (VHL). The search strategy included any study that evaluated IC through PR and CBCT, the strategy was made with indexed words (MeSH) and terms related to the IC, CBCT, and PR. The terms were combined and related through Boolean operators (AND / OR) for use in each bibliographic database. There was no restriction on language or date of publication. The databases search are in Supplement 1 

(http://www.medicinaoral.com/medoralfree01/aop/jced_61285_s01.pdf).

Gray literature was searched to include any additional work that met the eligibility criteria. The reviewers performed a manual search and reference lists of all selected studies and searched for theses and dissertations in OpenGrey, ProQuest, Brazilian digital library of theses and dissertations (BDTD-IBICT), and Google Scholar to find eligible works. Studies published until August 2023 were included.

-Study selection 

All studies collected from the different databases uploaded to Endnote Web software (www.myendnoteweb.com), where they were stored in a single folder and organized and verified to remove duplicated references. In addition, a manual search was performed to check that there were no duplications.

All study titles and abstracts were identified independently. The selection of the studies was performed by two calibrated reviewers (M.P.M. and C.M.F.R.), for determined the eligibility of studies based on the criteria described above. For potentially eligible studies, the full text was read and the studies were coded alphabetically and placed in a folder to facilitate further analysis. The discrepancy between the two reviewers about the eligibility of studies in both phases was resolved by discussing it with the third reviewer (I.R.F.R.B.).

-Data extraction and Data items 

The papers that met the inclusion criteria were examined independently by two reviewers (M.P.M. and C.M.F.R.). Data extraction was performed by these reviewers and any discrepancies were resolved by discussion with the third reviewer (I.R.F.R.B) until reached a mutual agreement.

For each of the selected studies, their main characteristics extracted for the synthesis of results using a standardized form in Microsoft Office Excel (Microsoft® Office). The information extracted included: first author, year of publication, geographic region, sample number, age and sex of the population, details of exposure (CBCT) and control (PR), examiners, methods used in CBCT/PR (Tables  Table 1- Table 2 cont.-1), measurements made of the studies and results (Tables  Table 3- Table 5). Only the information available in the articles was considered.

**Table 1:**
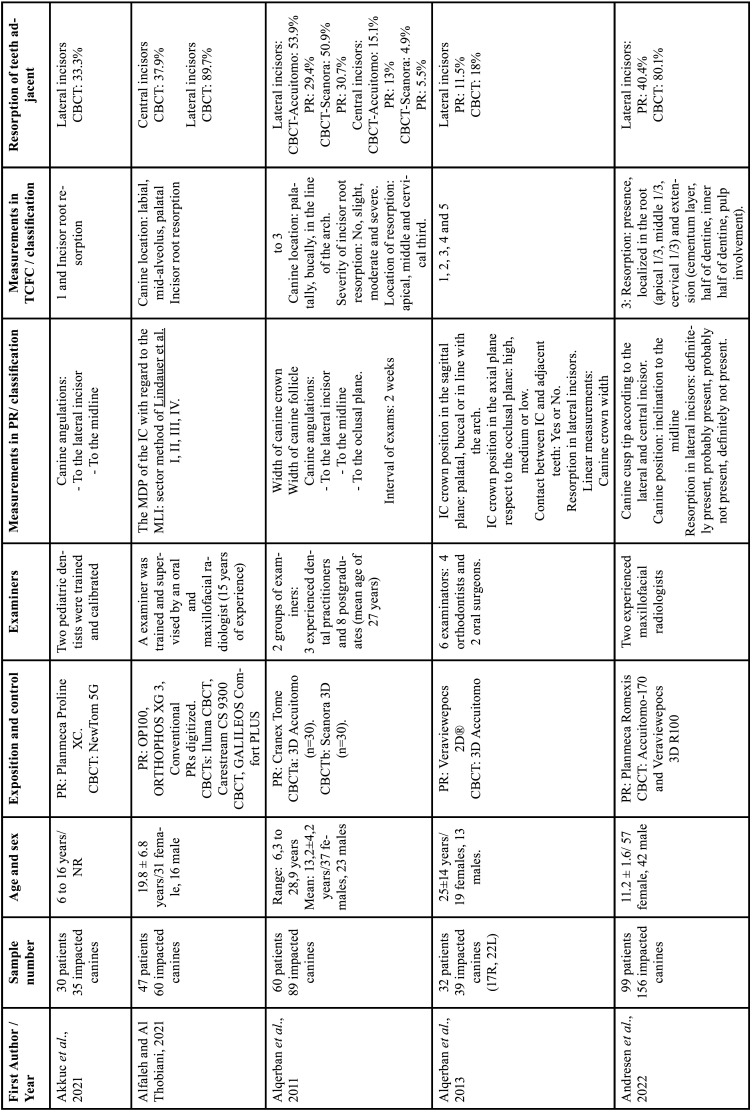
Details of included studies (in alphabetical order).

**Table 1 cont.**
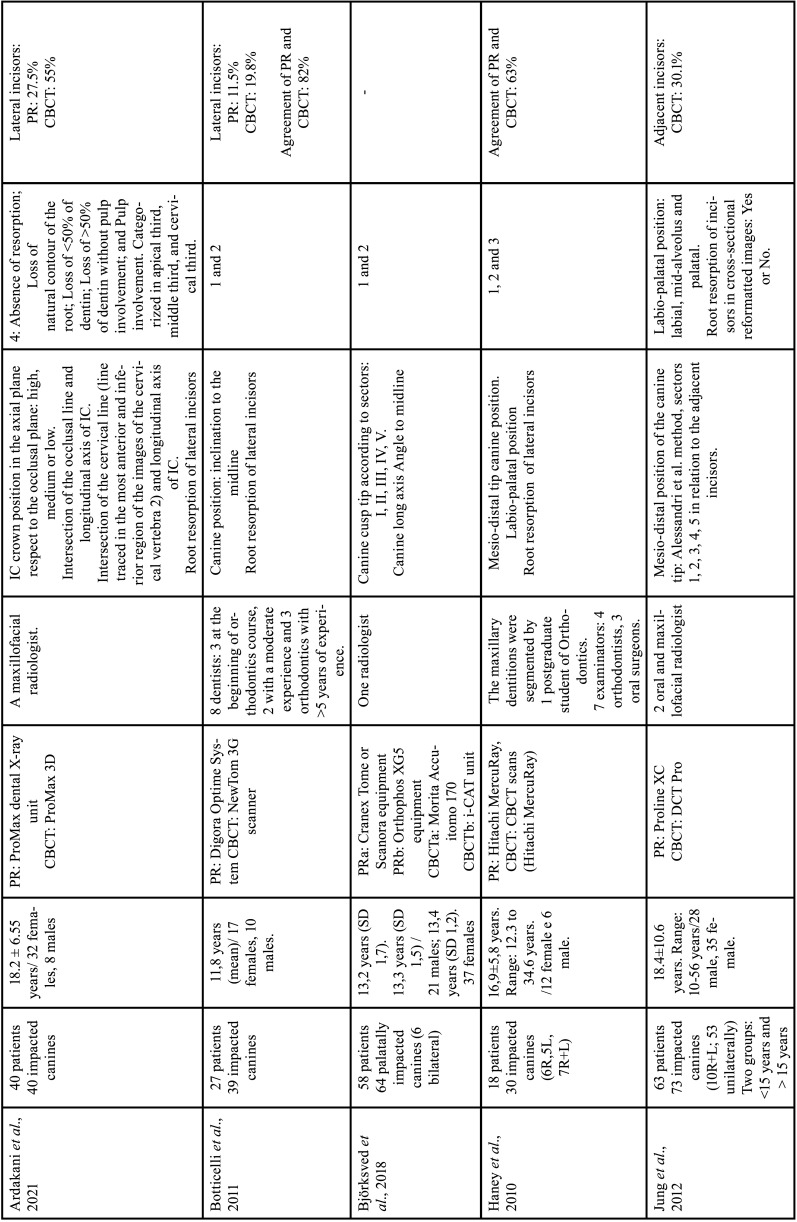
Details of included studies (in alphabetical order).

**Table 1 cont.-1:**
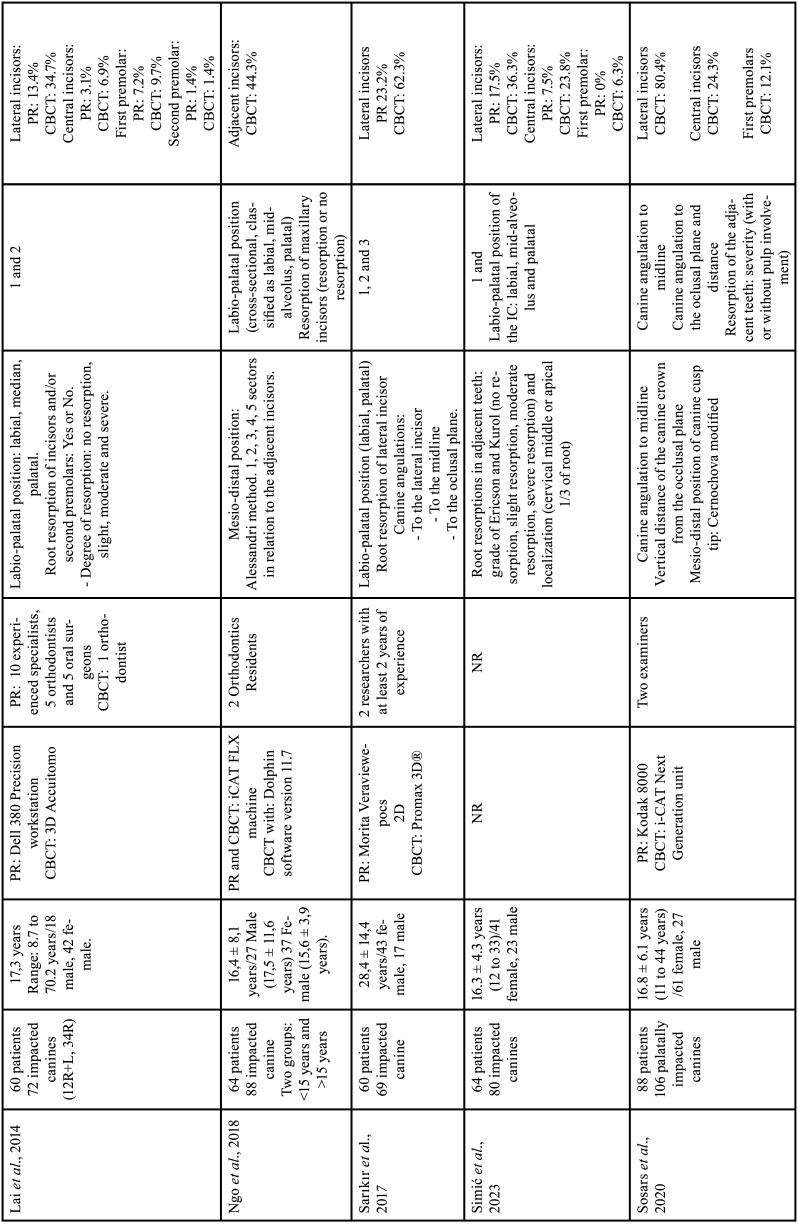
Details of included studies (in alphabetical order).

**Table 1 cont.-2:**
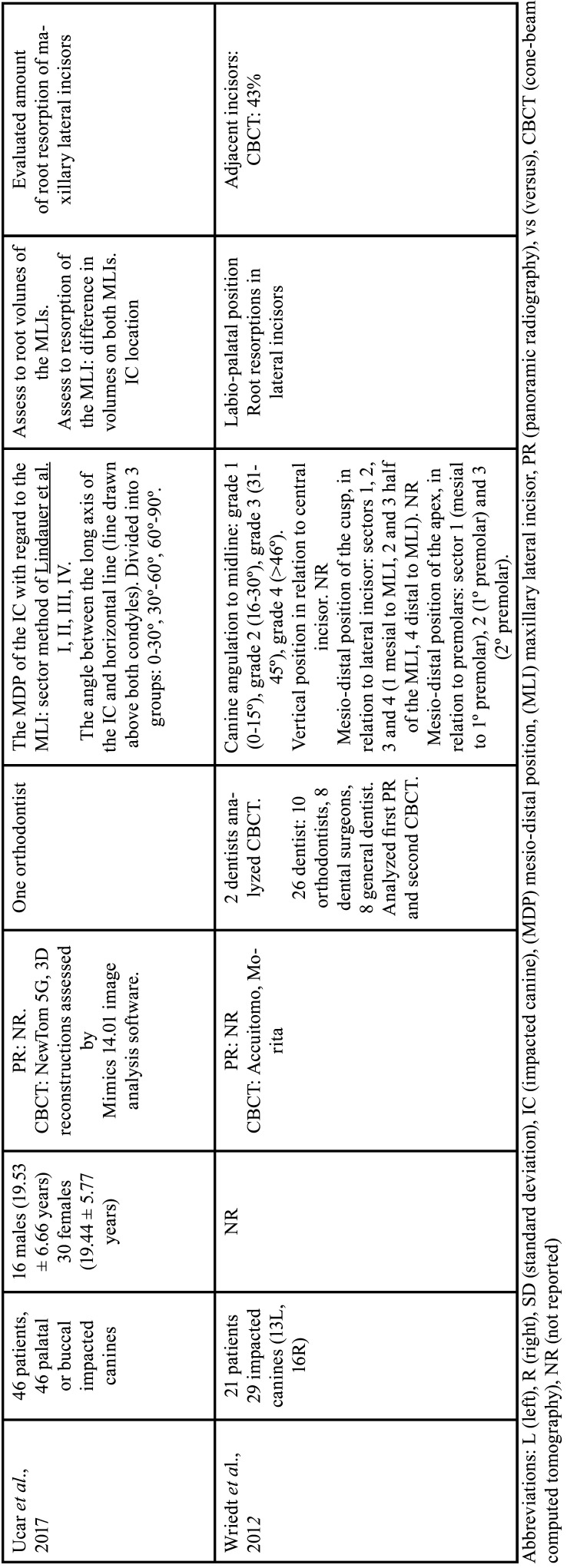
Details of included studies (in alphabetical order).

**Table 2:**
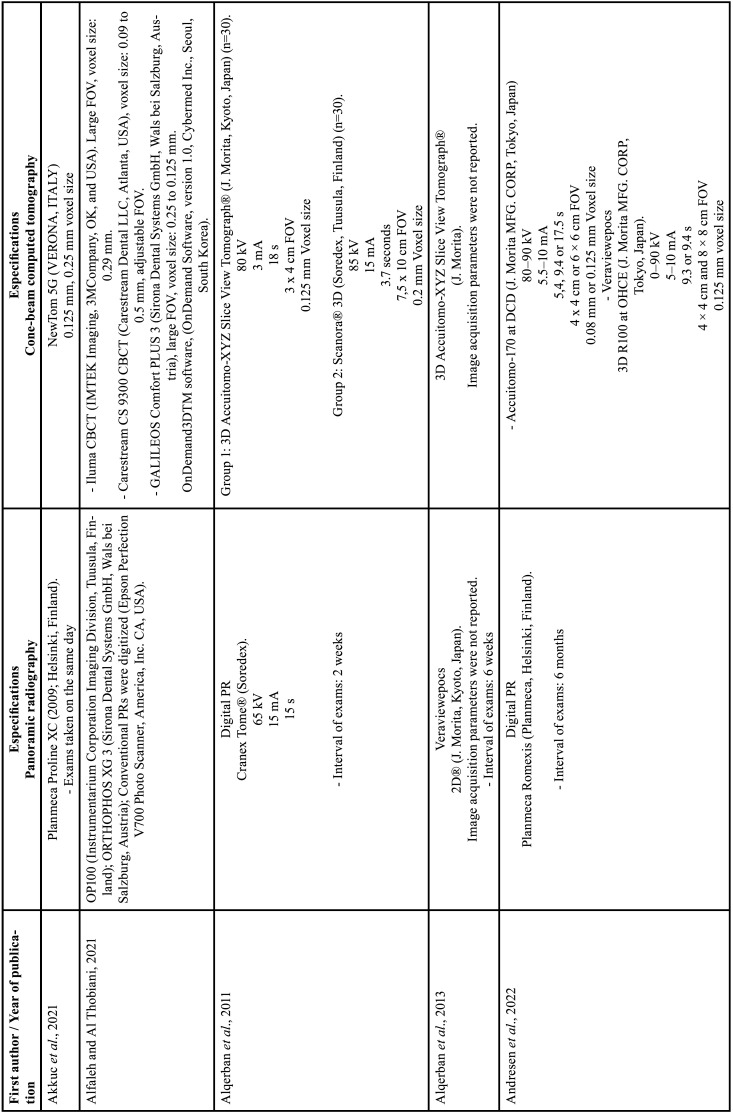
Specifications of image acquisition in panoramic radiography and CBCT.

**Table 2 cont.**
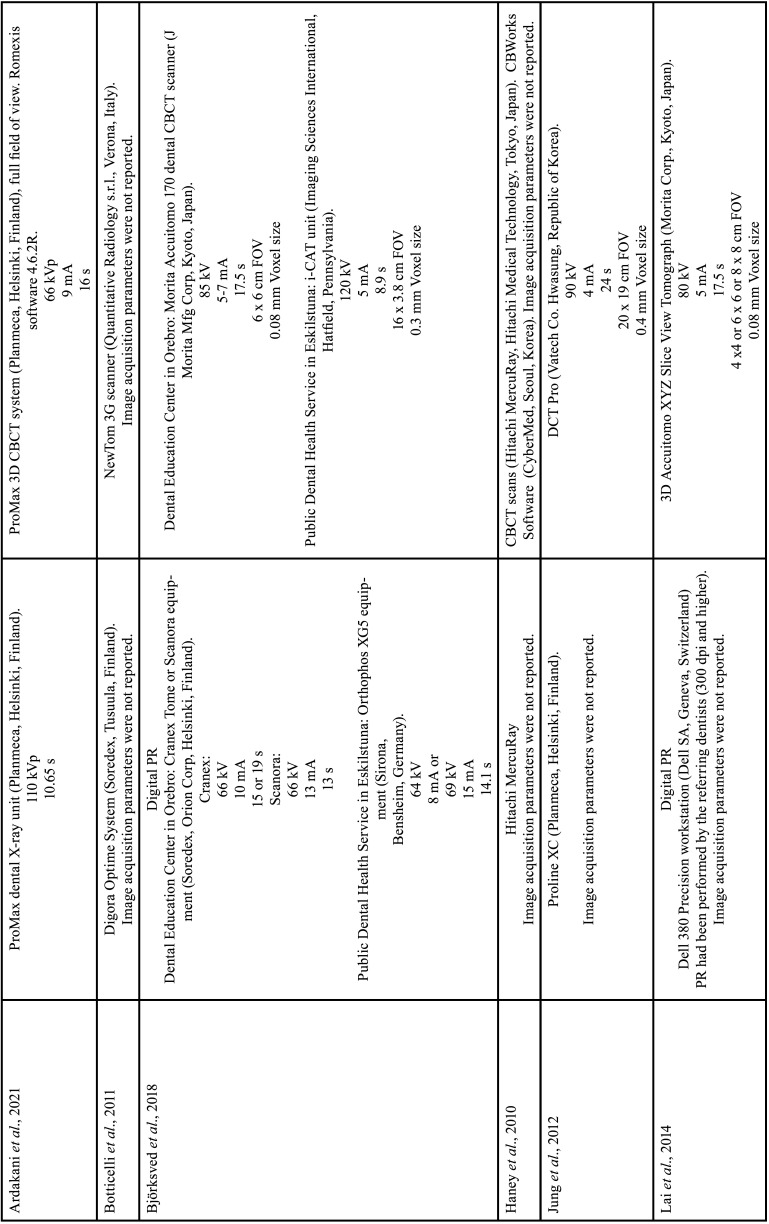
Specifications of image acquisition in panoramic radiography and CBCT.

**Table 2 cont.-1:**
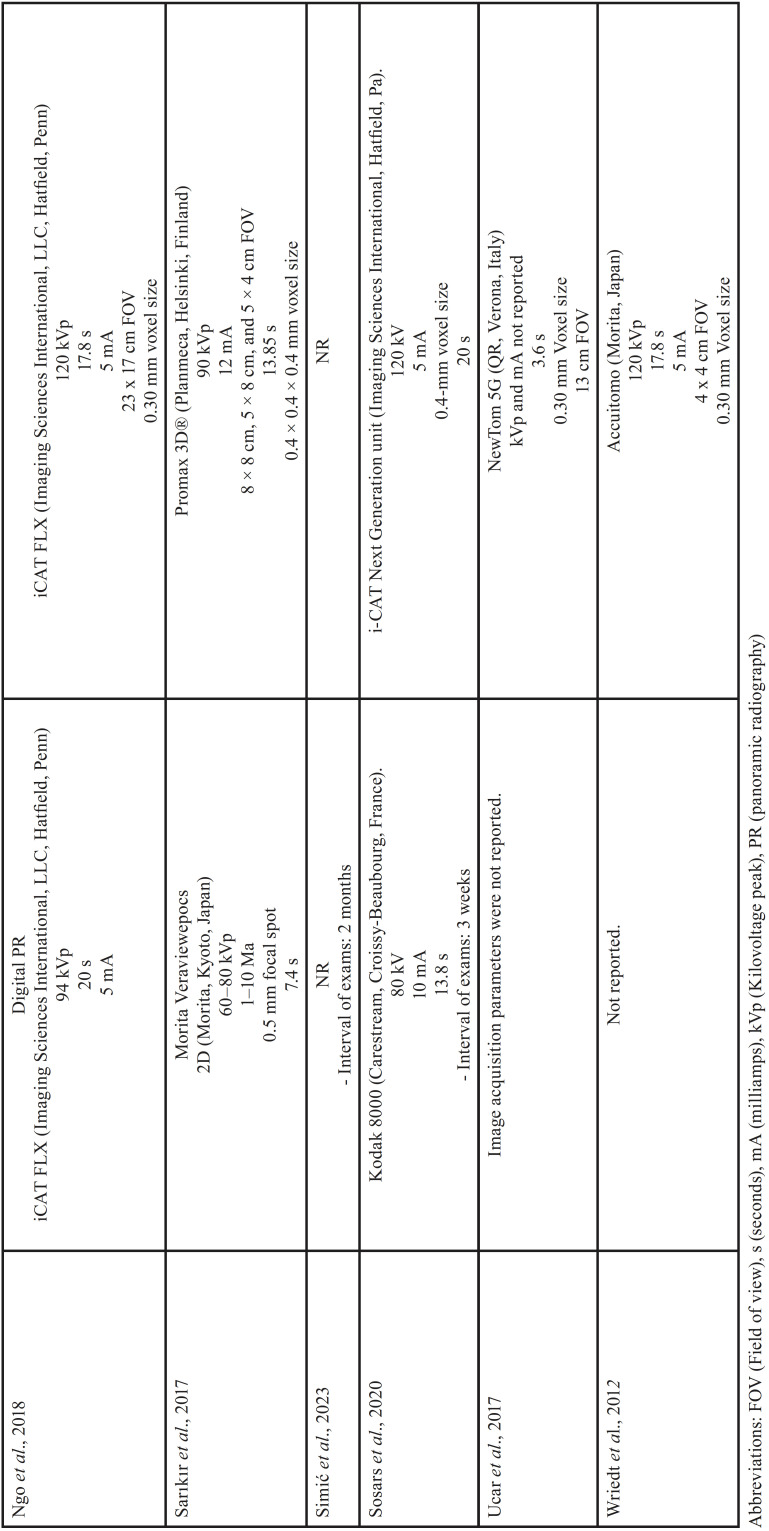
Specifications of image acquisition in panoramic radiography and CBCT.

**Table 3:**
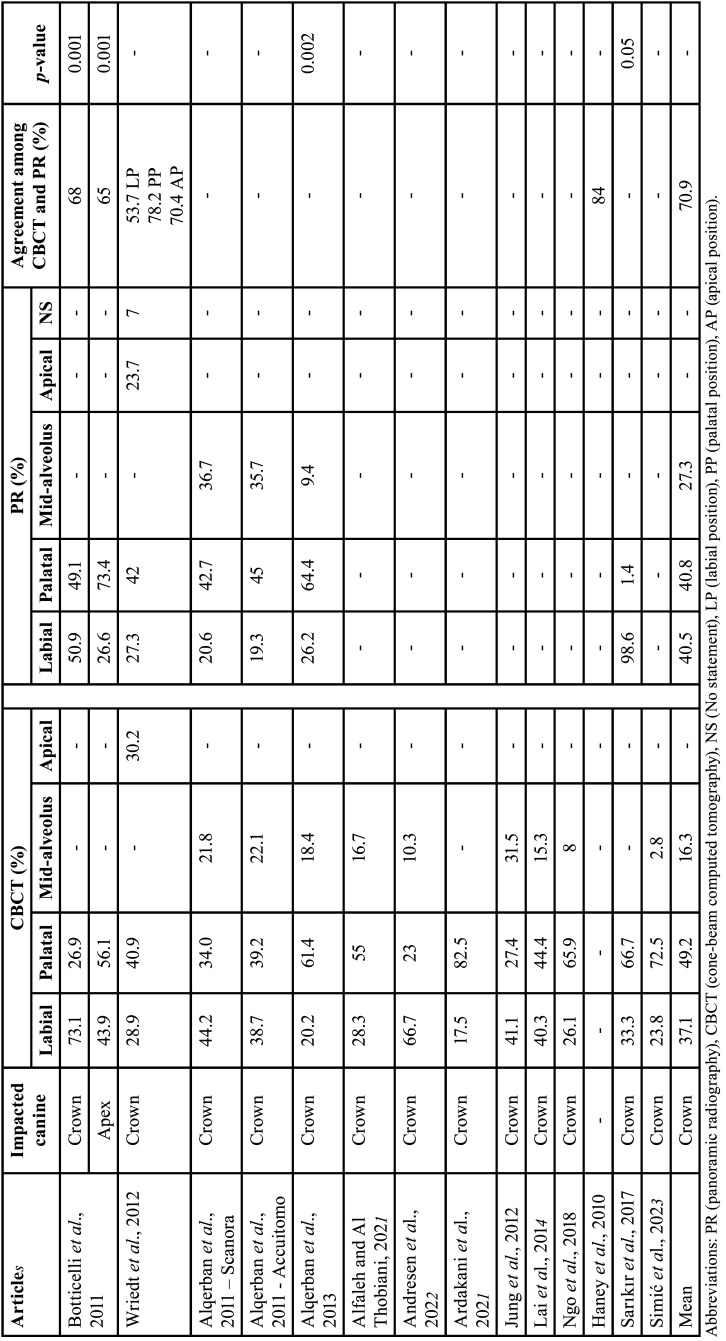
Data from studies that assessed the labio-palatal position of impacted canine, represented in percentages (%).

**Table 4:**
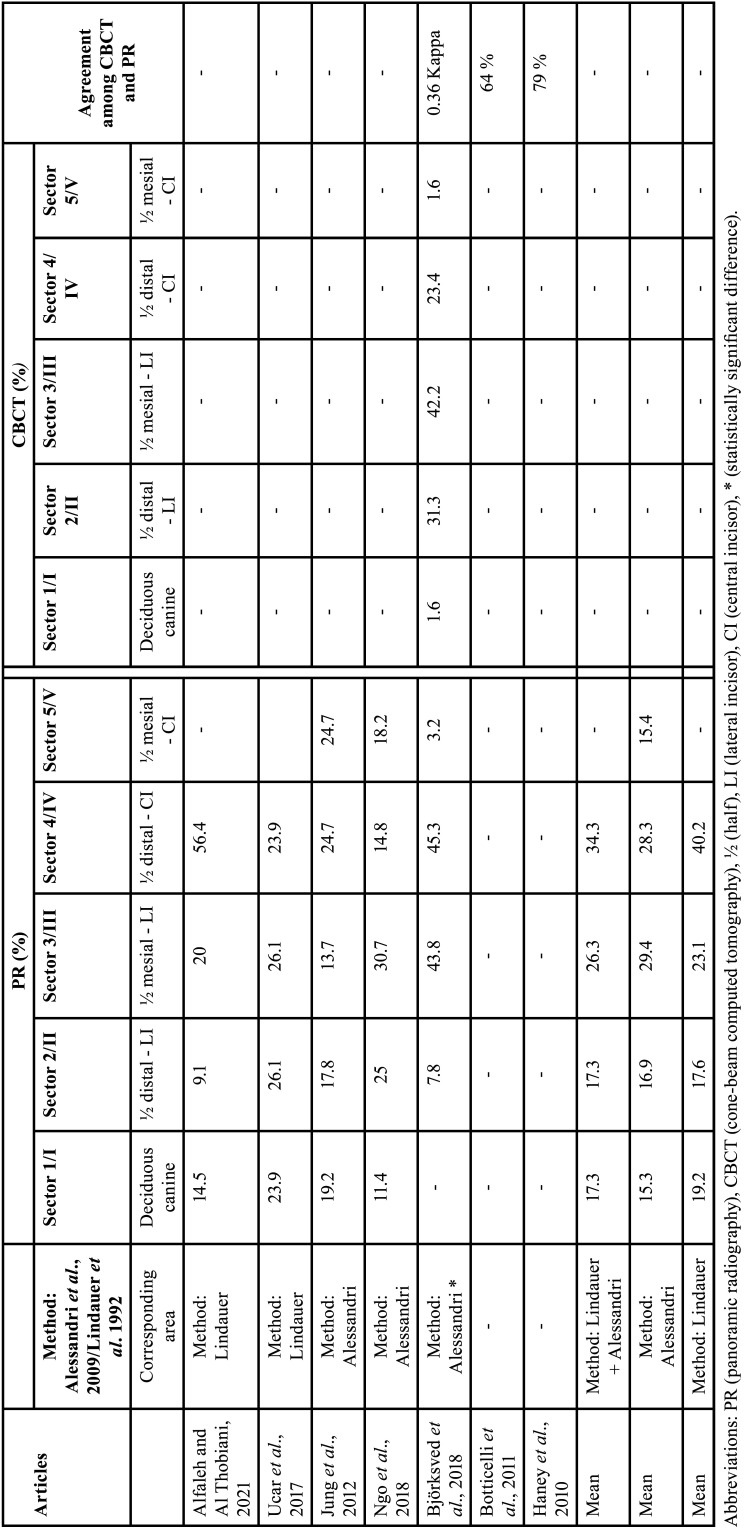
Data from studies that assessed the mesio-distal position of canine cusp tip, represented in percentages (%).

**Table 5:**
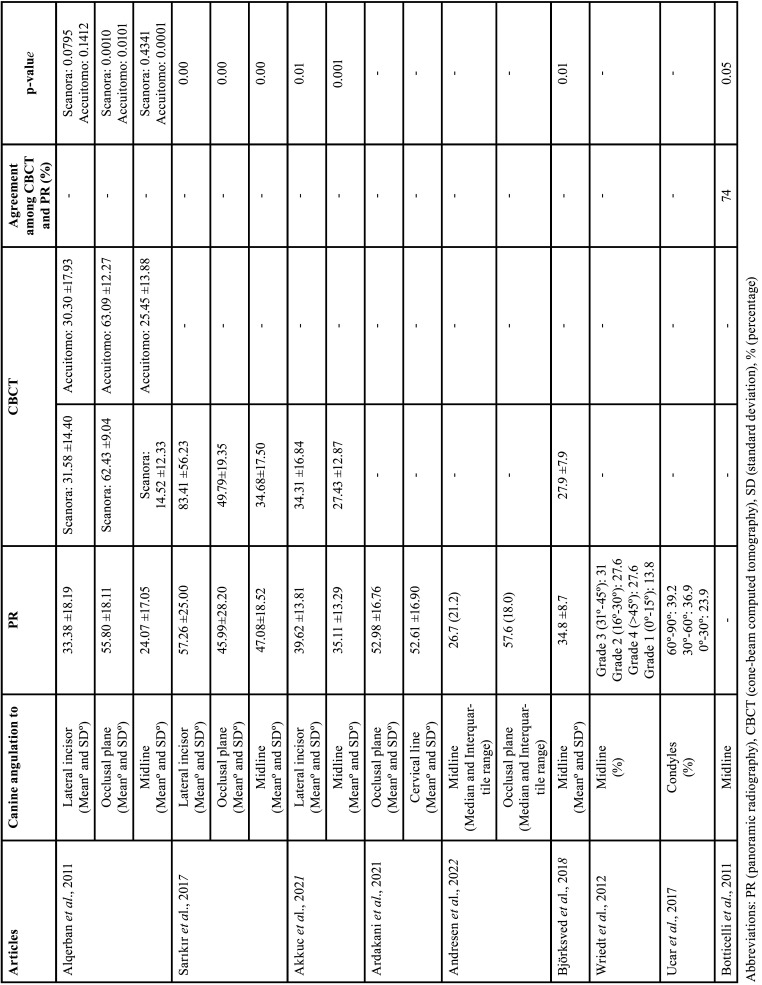
Impacted canine angulation with respect to the lateral incisor, midline, occlusal plane and condyles.

-Risk of bias of individual studies

We used the Appraisal tool for cross-sectional studies (AXIS tool) (15). The quality analysis was conducted through the use of 20 questions in the AXIS tool (about introduction, methods, results, discussion, and other) which were based on the following study aspects: quality of reporting, study design quality and possible introduction of biases. The reviewers assigned each guiding question one of three options: yes, no, do not know. Two reviewers (M.P.M. and C.M.F.R.) independently assessed the methodological quality of each study using the AXIS tool and any unresolved disagreement between the reviewers was resolved by a discussion with the third author (I.R.F.R.B.) (Table  Table 6- Table 6 cont.-2).

**Table 6:**
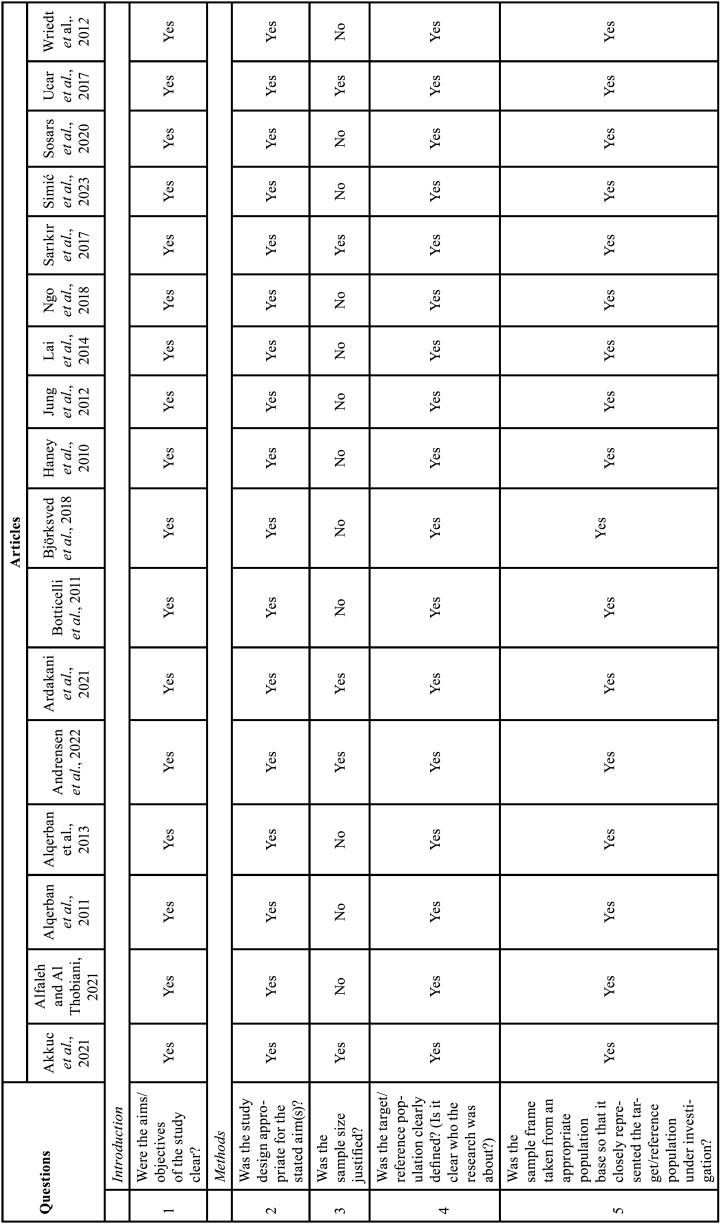
Risk of Bias Assessment. The answer to each question may be as follows: yes, no, do not know (15).

**Table 6 cont.**
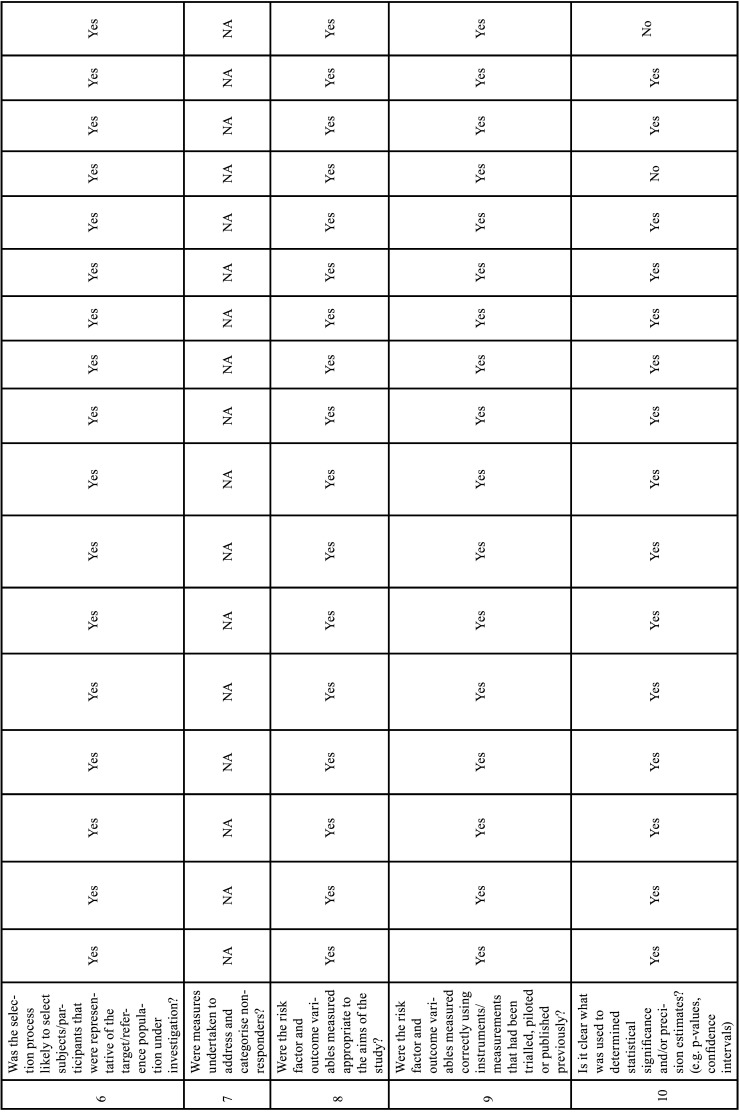
Risk of Bias Assessment. The answer to each question may be as follows: yes, no, do not know (15).

**Table 6 cont.-1:**
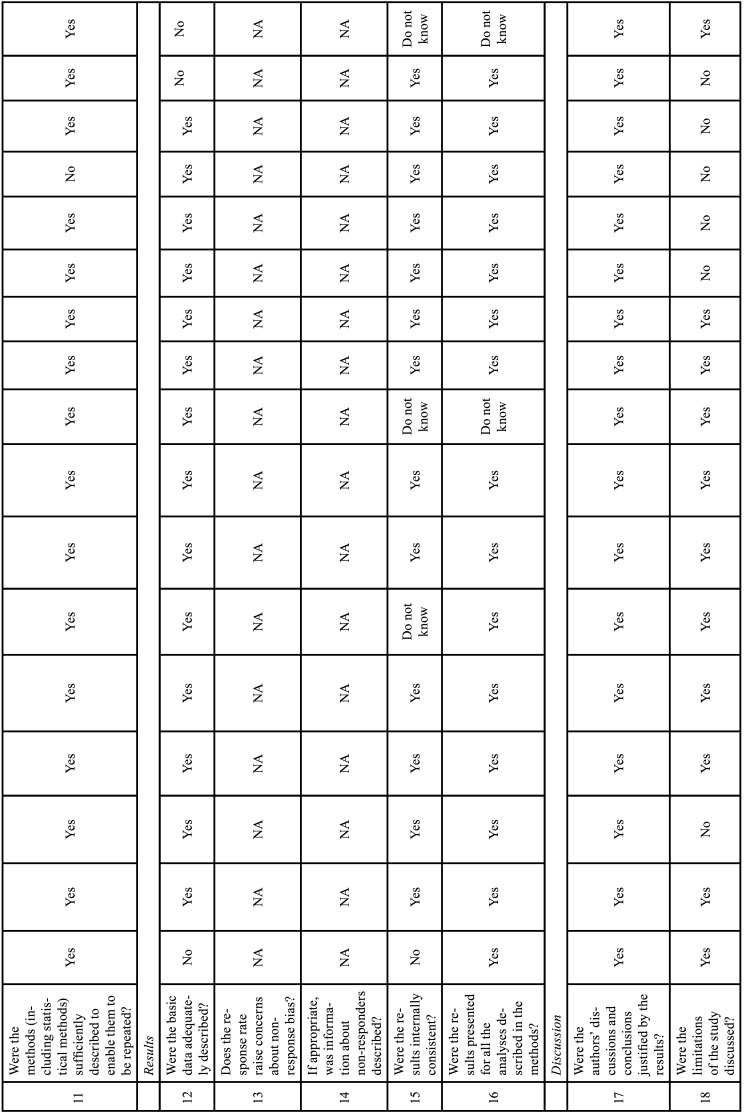
Risk of Bias Assessment. The answer to each question may be as follows: yes, no, do not know (15).

**Table 6 cont.-2:**
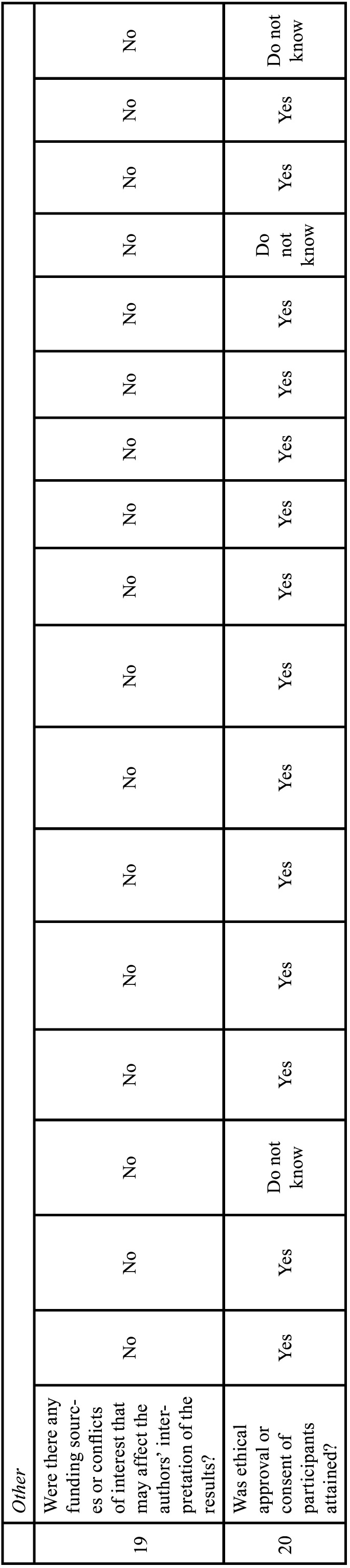
Risk of Bias Assessment. The answer to each question may be as follows: yes, no, do not know (15).

-Summary measures

Any type of prevalence outcome measurement of the IC position (labio-palatal position, mesio-distal position, vertical position, angulation with respect to the lateral incisor, midline, or occlusal plane), and resorption of the teeth adjacent to the IC was considered. In the case of the IC angulation, measures such as mean and standard deviation of the angulation in relation to the lateral incisor, occlusal plane, and midline were also considered.

-Synthesis of results 

A narrative synthesis and meta-analysis were carried out on some variables that had sufficient quantitative data. In order to reduce the heterogeneity between the studies, the results were separated according to the IC position considered by each study: labial-palatal, mesio-distal, vertical, and/or angulation of the IC. The result on the resorption of teeth adjacent to the IC was according to the type of tooth: lateral incisor, central incisor, or premolars.

To perform the meta-analysis, the Comprehensive Meta-Analysis software (Biostat; Englewood, NJ, USA) was used. The level of significance was 5%. The random effects model (16), and the Restricted maximum-likelihood was used as how random-effects estimator. The heterogeneity found in the meta-analysis of IC angulation to the midline, occlusal plane, lateral incisor was high. The heterogeneity of resorption of the lateral incisor and premolars adjacent to the IC was high, whereas in central incisor, it was low. In the meta-analyzes of labial-palatal position, there was low heterogeneity in mid-alveolus position, whereas it was high in labial and palatal positions.

## Results

-Study selection

**Figure 1:**
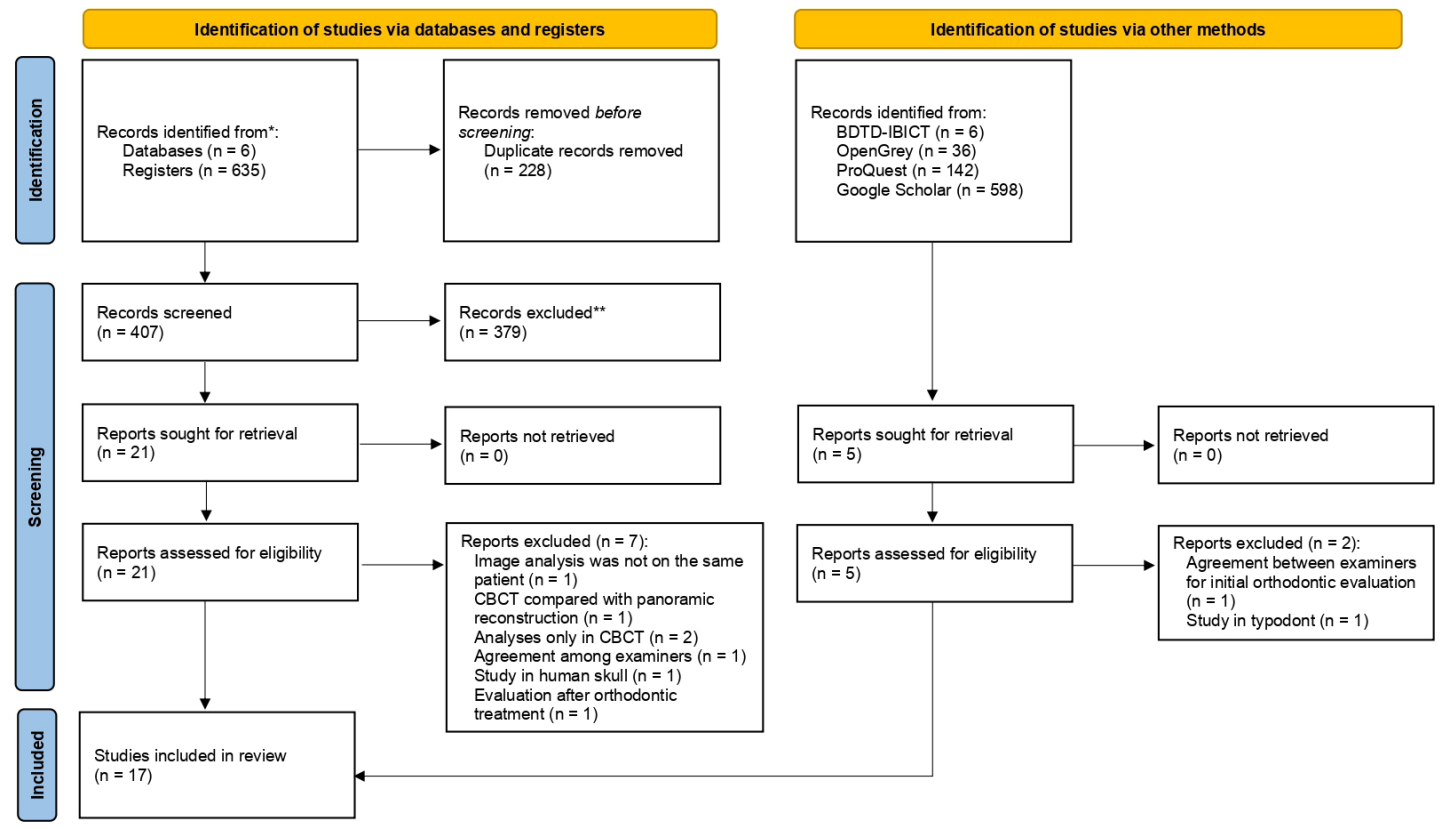
Flowchart of study selection for qualitative and quantitative syntheses.

A total of 635 studies were collected after applying the initial search strategies in databases. After excluding the repeated records, 407 articles remained. In the gray literature, 782 records were found and only 5 studies were potentially eligible. After submitting the articles to the eligibility criteria, twenty six studies were selected for full reading, twenty one from the databases and five from the gray literature. In total nine studies were excluded: one because the image analysis was not in the same patient (17), one because the CBCT was compared with the panoramic reconstruction (18), two because the analyzes only used CBCT (9,19), one because it only reported data from the agreement of examiners about the location of the impacted canine and resorption of adjacent teeth (20), one because assessed the agreement between examiners for initial orthodontic evaluation, answering questionnaires (21), one study because it was performed on typodonts (22), one study because it was performed on deceased human skulls (23), and one because evaluated after orthodontic treatment (24). After these exclusions seventeen articles were considered eligible for this study (9,12,13,25-38) (Fig. 1).

-Study characteristics 

The publication period for the ten included studies was from 2010 to 2023 and the studies were carried out in Leuven, Belgium (12,28), Aarhus, Denmark (13), Eskilstuna, Sweden (30), Busan , Republic of Korea (26), Bern, Switzerland (9), San Francisco, USA (25), New York, USA (31), Kayseri, Ankara, Malatya, Turkey (29,32,38) and Rhineland Palatinate, Germany (27), Riga, Latvia (37), kosovska Mitrovica, Serbia (36), Rafsanjan, Iran (35), Bergen, Norway (34), Riyadh, Saudi Arabia (33) (Table 1- Table 1 cont.-2).

Cross-sectional observational studies whose samples were assessed using PR and CBCT of the same patient, to describe the position of the IC and resorption of adjacent teeth were included. In total, 877 patients were included in the seventeen studies, with a total of 1115 impacted upper canines. The mean age of patients was 17.6 years, there were 531 women and 295 men. One study not reported the age and gender of 21 patients (27). One study reported only the range of 6 to 16 years in 30 patients and did not report gender (32). Table 1- Table 1 cont.-2 summarizes the descriptive characteristics of the seventeen studies.

-Risk of bias within studies 

The quality assessments of the individual studies are listed in Table  Table 6,  Table 6 cont.-2. All studies included in this systematic review were cross-sectional studies. The studies adequately addressed the study design, the quality of the reporting of results, and the risk of bias. However, only five studies justified the sample size (29,32,34,35,38), two studies in the methodology did not report the type of statistical analysis used (27,36), one study did not describe the equipment used in RP and CBCT, image acquisition parameters, experience and number of examiners, images considered to perform the analyses (36), two studies did not report data on gender and mean age (27,36), two studies did not report RP acquisition data (27,29). One study was divided into 4 phases, in the fourth phase they proposed to compare PR and CBCT performed on the same day, however, the location of IC was not described in detail by sectors as described in the methodology, they only presented data of mean, SD, median, min, max (32). Four studies did not show results that were internally consistent (25,27,32,35). The limitations were not discussed in six studies (12,29,31,36-38) and in three studies it was not clear whether the study was ethically approved (12,27,36).

-Results of individual studies

The primary outcome was the position assessing of IC and resorption of adjacent teeth through panoramic radiography and CBCT.

Impacted upper canine position

The seventeen studies included in this review assessed IC position in the following ways.

• Labio-palatal position / sagittal plane, classified as labial, palatal or mid-alveolus (9,12,13,25-28,31,33-36,38) 

• Mesio-distal position (13,25,26,29-31,33)

• Vertical position / axial plane, classified as grade 1, 2, 3, 4 (13) and high, medium, low (28)

• Angulation with respect to the lateral incisor, midline or occlusal plane (12,13,27,29,30,32,34,35,38) 

-Resorption of teeth adjacent to the impacted upper canine

Fifteen studies evaluated the occurrence root resorption of adjacent teeth (9,12,13,25-28,31-38). Root resorption of teeth adjacent to the IC was more frequently detected with CBCT (29.9%) than with PR (15.2%) 

Eleven studies evaluated the resorption of lateral incisors. Three studies evaluated only with CBCT, finding 67.8% resorption (32,33,37). Eight studies compared both methods, finding greater reabsorption in CBCT (45.7%) compared to PR (22.8%) (9,12,13,28,34-36,38).

Five studies evaluated the resorption of central incisors. Two studies evaluated only with CBCT, finding 31.1% resorption (33,37). Three studies compared both methods, finding greater reabsorption in CBCT (12.7%) compared to PR (7.3%) (9,12,36).

Three studies evaluated resorption in first premolars. One of them evaluated only on CBCT, finding 12.1% reabsorption. Two studies compared both methods, finding greater reabsorption in CBCT (8%) compared to PR (3.6%) (9,36). A single study reported resorption in second premolars, finding 1.4% of cases with both exams (9).

Three studies evaluated resorption of the adjacent incisors on CBCT, presenting 30.1%, 43%, and 44.3% of resorption cases (26,27,31). The agreement of PR and CBCT evaluation respect to root resorption of adjacent incisors varied from 63% (25) to 82% (13).

-Labio-palatal position 

Six studies evaluated the labio-palatal position of the IC (crown or apex) through CBCT and PR (9,13,25,27,28,38). Seven studies evaluated the labio-palatal position only by CBCT (9,26,31,33-36) (Table 3).

In one study, this was evaluated according to the concepts of horizontal amplification, which is determined by the position of the object within the image layer. If the crown of the IC was magnified in the image, it indicated the palatine position of the tooth. If the crown was narrow, it indicated the labial position of the tooth (28). The other study used only panoramic radiography for this evaluation, however, the authors did not specify the evaluation parameters (9).

In CBCT, the crown of the IC is most often found in the palatal position (49.2%), followed by the labial position (37.1%), and mid-alveolus position (16.3%). In PR, the IC labial and palatal position is the most frequent (40.5% and 40.8%), followed by the mid-alveolus position (27.3%). The labio-palatal position of the apex was determined in one study. With CBCT the palatal position of the apex occurred in 56.1% of cases and labial position occurred in 43.9%. In PR, palatal position of the apex occurred in 73.4% cases and labial position in 26.6% (13).

The agreement between PR and CBCT regarding the labio-palatal position of the IC was determined by three studies (70.9%) (13,25,27).

-Mesio-distal position 

Seven studies evaluated the mesio-distal position of the IC through CBCT and PR (13,25,26,29-31,33). Two studies evaluated the agreement between PR and CBCT regarding the mesio-distal position. One study evaluated the position of canine cusp tip and classified it as mesial, distal or direct. Resulting in 79% agreement between exams (25). Another study evaluated the mesio-distal position of the apex, classified as Grade 1 (above the region of the canine), Grade 2 (above the first premolar) and Grade 3 (above the second premolar). In the PR exams, the IC position was most frequently found in the first premolar region and in the CBCT a larger spread was observed. There was a 64% agreement between the exams, with PR indicating less variation in the position of the IC apex (*p* = 0.001) (13) (Table 4).

Two studies evaluated the mesio-distal position of IC cusp tip using PR and by the sector method of Lindauer *et al*. (39): sector I (region distal to the lateral incisor), sector II (distal half of the lateral incisor), sector III (mesial half of the lateral incisor) and sector IV (region mesial to the lateral incisor). The result showed that the IC was more frequently found in sector II (26.1%) and sector III (26.1%), followed by sector I (23.9%) and sector IV (23.9%) (29). The other study showed that the UC was more frequently found in sector IV (56.4%), followed by sector sector III (20%), sector I (14.5%) and sector II (9.1%) (33).

Three studies evaluated the mesio-distal position of canine cusp tip (26,30,31) by the sector method of Alessandri *et al*. (40): sector 1 (corresponding to the deciduous canine-present or absent), sector 2 (the distal half of the lateral incisor), sector 3 (the mesial half of the lateral incisor), sector 4 (distal half of the central incisor) and sector 5 (mesial half of the central incisor to the midline). In the PR evaluation, the IC was found more frequently in sectors 3 (29.4%) and 4 (28.3%), followed by sectors 2 (16.9%), 5 (15.4%), and 1 (15.3%). One study evaluated the mesio-distal position through CBCT and PR, showing that PR classified the IC in higher sectorial values compared to the analysis with CBCT (*p* <0.01; kappa 0.36) (30).

-Vertical position 

The vertical position of the IC in relation to the axial plane or occlusal plane was evaluated in two studies (13,28). PR shows a higher position compared to CBCT.

The first study classified the vertical position by Stivaros and Mandall method (41), respective to the adjacent upper incisor as grade 1 (below the cemento-enamel junction (CEJ), grade 2 (above the CEJ, but below the half way point of the root), grade 3 (half or more apical from the root, but below the apex) and grade 4 (above the apex). There were eight evaluators, resulting in a 66% agreement between PR and CBCT (*p* = 0.013), with PR showing a higher vertical position, being more apical to the lateral incisor (13). The second study assessed the vertical position in relation to lateral incisor root (LIR) and classified as high (apical third of LIR), medium (middle third of LIR), and low (coronal third of LIR). The results showed a statistically significant difference between PR and CBCT (*p* = 0.005). In PR there 51.7% of cases were classified as medium, followed by 30.3% high and 18% low. The CBCT saw 43.6% medium, followed by 29% high and 27.4% low (28).

-Canine Angulation 

Nine studies analyzed the IC angulation through CBCT and PR (12,13,27,29,30,32,34,35,38] (Table  Table 5).

Seven studies analyzed IC angulation to the midline (12,13,27,30,32,34,38). The average was 33.6° in PR and 26° in CBCT. One study found 74% agreement between the PR and CBCT (*p*>0.05) (13). Two studies measured only CBCT (27,34). Four studies that compared CBCT and PR found a statistically significant difference between measurements (*p*<0.05) (12,30,32,38).

Three studies evaluated IC angulation to the lateral incisor with two lines drawn along the long axis of the IC and lateral incisor. The average PR was 43.4° and the CBCT was 44.9°. Two studies found a statistically significant difference between CBCT and PR means (*p*<0.05) (32,38) and one study found no difference between the methods (*p*>0.05) (12).

Four studies evaluated the IC angulation to the occlusal plane, where two lines were drawn along the long axis of the IC and the occlusal plane. Two studies measured only CBCT, obtaining an angle of 52.98 to 57.6° (12,35). Two studies that compared CBCT (58.4°) and PR (50.9°) found a statistically significant difference between measurements (*p*<0.05) (12,38).

One study evaluated IC angulation to the line between both condyles only in PR, with two lines drawn along the long axis of the IC and a line drawn between superior points of both condyles (29).

-Synthesis of results 

The meta-analysis was performed with eleven studies (9,12,13,27,28,30,32,34-36,38).

**Figure 2:**
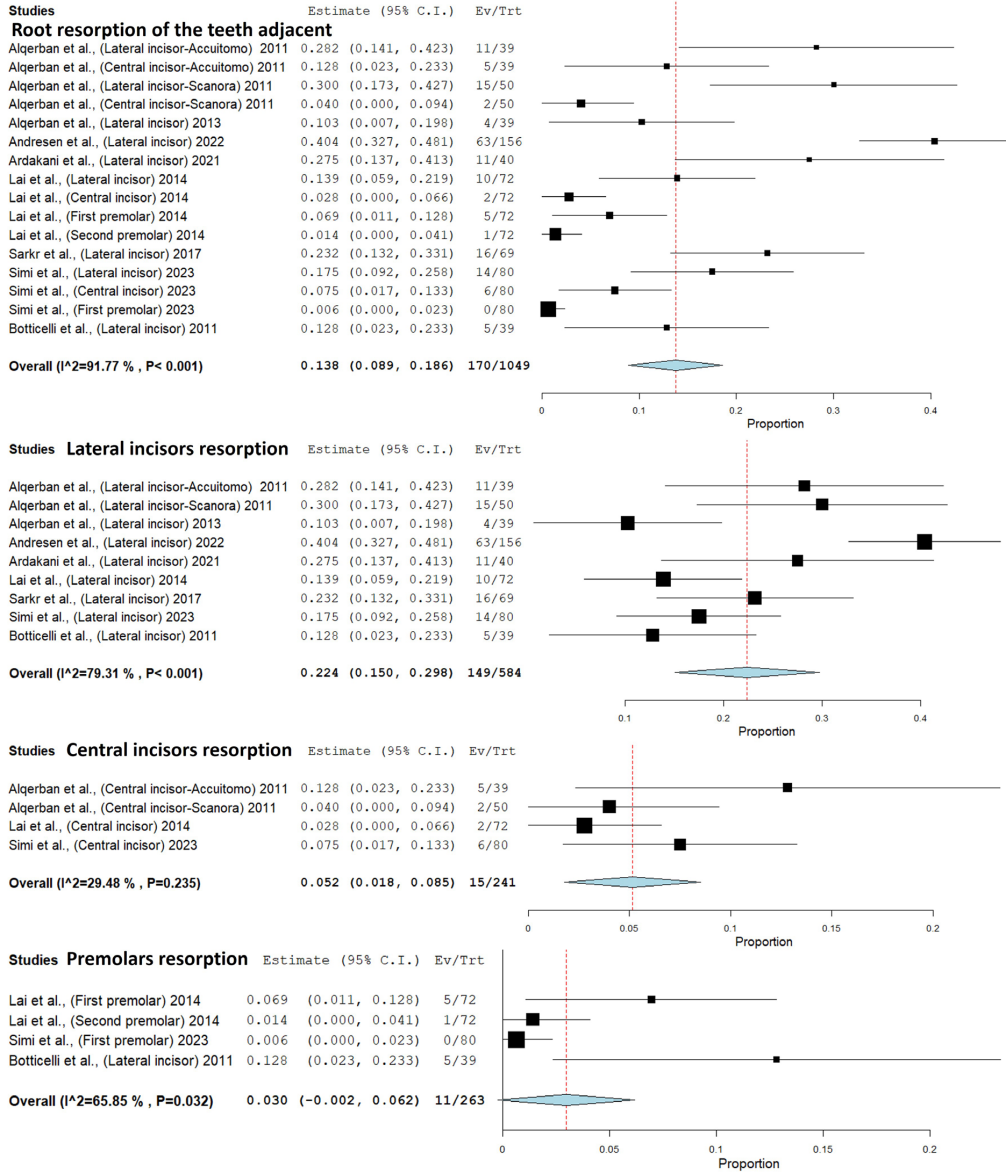
Comparison between panoramic radiography and cone-beam computed tomography, in the number of cases detected with resorption of the teeth adjacent to the IC.

Eight studies (9,12,13,28,34-36,38) were eligible for the meta-analysis of root resorption of the teeth adjacent to the IC. The results showed that in PR there was 86% less chance of finding resorption of the teeth adjacent to the IC when compared with CBCT. Thus, CBCT detected a larger number of cases of resorption of teeth adjacent to the IC (*p*<0.001) (Confidence interval 95%, 0.089 - 0.186; heterogeneity: Q value 182.313; I2 91.772%; Tau squared 0.008; *P*-value 0.001; Odds Ratio [OR] value: 0.138; n=1049). In the analysis of subgroups, CBCT showed 78% more cases of lateral incisor resorption than PR (*p*<0.001) (Confidence interval 95%, 0.150 - 0.298; heterogeneity: Q value 38.665; I2 79.31%; Tau squared 0.010; *P*-value 0.001; Odds Ratio [OR] value: 0.224; n=584). CBCT showed 95% more cases of resorption of central incisor than PR (*p*<0.001) (Confidence interval 95%, 0.018 - 0.085; heterogeneity: Q value 4.254; I2 29.477%; Tau squared 0.000; *P*-value 0.235; OR value: 0.052). CBCT showed 97% more cases of premolars resorption than PR (*p*=0.032) (Confidence interval 95%, -0.002 - 0.062; heterogeneity: Q value 8.785; I2 65.85%; Tau squared 0.001; *P*-value 0.032; OR value: 0.032) (Fig. 2).

**Figure 3:**
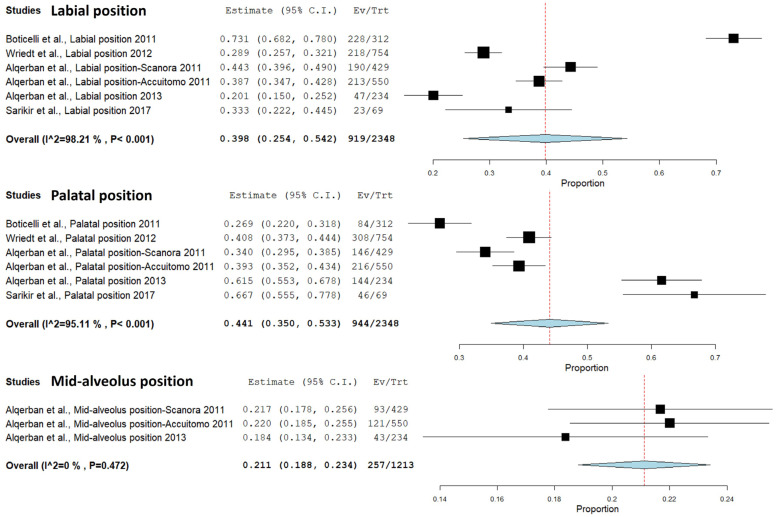
Comparison of the labio-palatal position through panoramic radiography and cone-beam computed tomography.

A meta-analysis of the IC position through PR and CBCT was performed in five studies (12,13,27,28,38). CBCT showed 60% more cases of labial position than PR (*p*<0.001) (Confidence interval 95%, 0.254 - 0.542; heterogeneity: Q value 279.387; I2 98.21%; Tau squared 0.032; *P*-value 0.001; Odds Ratio [OR] value: 0.398). Additionally, CBCT demostrated 56% more cases of palatal position than PR (*p*<0.001) (Confidence interval 95%, 0.350 - 0.533; heterogeneity: Q value 102.189; I2 95.107%; Tau squared 0.012; *P*-value 0.001; Odds Ratio [OR] value: 0.441). Moreover, CBCT exhibited 79% more cases of mid-alveolus position than PR (*p*<0.001) (Confidence interval 95%, 0.188 – 0.234; heterogeneity: Q value 1.502; I2 0%; Tau squared 0.000; *P*-value 0.472; Odds Ratio [OR] value: 0.221) (Fig. 3).

**Figure 4:**
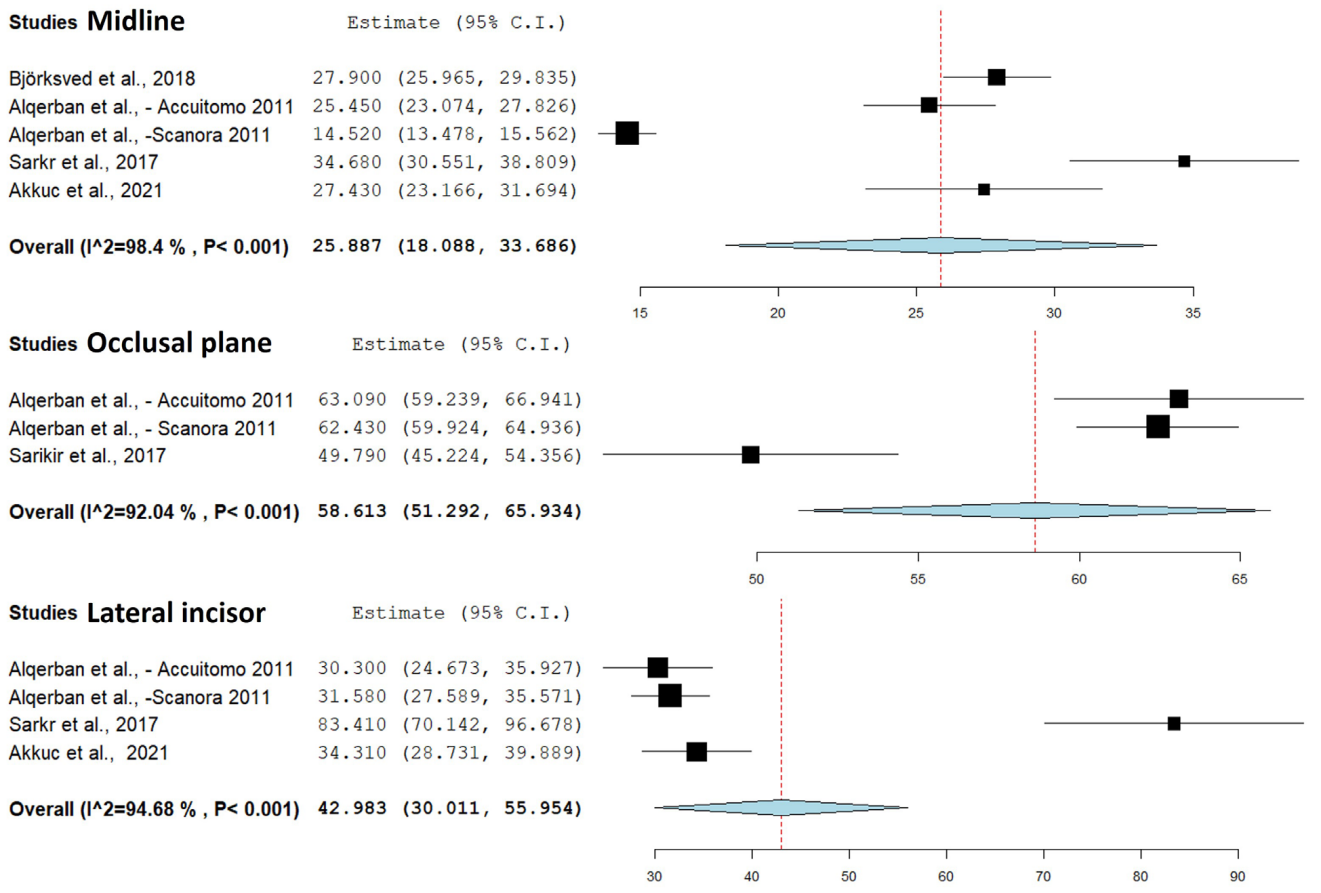
Comparison of the IC angulation to the midline, occlusal plane, and lateral incisor through panoramic radiography and cone-beam computed tomography.

The IC angulation (to the midline, occlusal plane, lateral incisor) meta-analysis was carried out in four studies (12,30,32,38). A significant statistical difference was observed between PR and CBCT. For IC angulation to the midline, four studies revealed a substantial difference (*p*<0.001), with CBCT showing a smaller and more accurate angle than PR (Confidence interval 95%, 18.008 – 33.686; heterogeneity: Q value 249.364; I2 98.396%; Tau squared 76.834; *P*-value 0.001). In the meta-analysis of IC angulation to the occlusal plane, two studies indicated a smaller angle in PR compared to CBCT (*p*<0.001) (Confidence interval 95%, 51.292– 65.934; heterogeneity: Q value 25.141; I2 92.045%; Tau squared 38.274; *P*-value 0.001). Similarly, for IC angulation to the lateral incisor, three studies demonstrated a smaller angle in PR compared to CBCT (*p*<0.001) (Confidence interval 95%, 30.011– 55.954; heterogeneity: Q value 56.348; I2 94.676%; Tau squared 160.096;*P*-value 0.001) (Fig. 4).

## Discussion

The initial assessment of ICs is often performed using PR images. However, this is often complemented with CBCT as this helps to recognize cases of IC with ankylosis (14.8%), dilaceration of the root (17.9%), resorption of adjacent teeth (14.8%) or odontoma (1.9%) (42). This complementary exam assists in diagnosis and is important in the preoperative analysis for orthodontists and surgeons, as they need precision in identifying the IC position to generate an appropriate treatment plan (6). This study used a systematic review to determine whether CBCT is better than PR at assessing the position of the unerupted upper canine and its effects on adjacent teeth.

Therefore, PR should be complemented with CBCT following the principle of As Low As Reasonably Achievable (ALARA) and As low as diagnostically acceptable (ALADA), according to European guidelines for radiation protection (43). To reduce the radiation dose, field-of-view (FOV) can be reduced. One study showed that the FOV required for IC was smaller than the smallest FOV offered by CBCT devices. Thus, reduced FOV to promote radiation safety is recommended (44).

CBCT was more effective than PR in assessing cases that are difficult to diagnose in the initial assessment of IC (45). The evaluation of IC by CBCT can provide more accurate angle measurements, linear measurements and better evaluation of cases with resorption of adjacent teeth (46). Our study agrees with these findings; the results showed that CBCT provided better results compared to PR with regards to identification of the IC location and resorption of adjacent teeth.

The presence of root resorption of teeth adjacent to the IC was detected in 15.2% of cases using PR and 29.9% using CBCT. The agreement between the exams was on average 72.5%. This result shows that CBCT detects more cases of root resorption, detecting almost double the cases seen with PR. Root resorption is more frequent when the IC is vertically above the apex of the lateral incisor root and close to midline (19). When this pathology is present, it can affect the treatment plan, if the reabsorption is very severe, tooth extraction would be indicated. Therefore, in these cases CBCT can contribute to accurate and timely diagnosis, and thus allow clinicians to carry out an appropriate treatment (6).

Regarding the labiopalatal position of the IC crown, in the evaluation of CBCT in two studies, they found that the IC was found most commonly in the labial position (57.1%) (13,26). However, this result differed from the findings of our study that found that IC was found most commonly in the palatal position (49.2%), followed by the labial position (37.1%), and mid-alveolus position (16.3%). In RP, the palatal and vestibular position was found with similar frequency (40.8% and 40.5%), and a higher frequency of mid-alveolar cases compared to CBCT (27.3%). The agreement between PR and CBCT on average was 70.9%. Therefore, CBCT appears to be more effective in evaluating the IC position. This result is due to the overlap of structures in the PR.

In the mesio-distal position of the IC, only one study evaluated the position of the apex of the IC, being more frequently found in the first premolar region (13). Five studies determined that the cusp tip of IC is most frequently found in sectors 3 and 4. Therefore, the position of the IC crown was commonly found in the sectors corresponding to the central and lateral incisor (26,29-31,33). Due to this, these are the teeth that present the more cases of root resorption. Furthermore, the agreement between CBCT and PARA was 71.5%. This shows that CBCT is better at evaluating the mesio-distal position of the IC.

Furthermore, the angulation of the IC with respect to the midline is greater when evaluated with the PR compared to CBCT (12,30,32,38). The angulation of the IC with respect to the occlusal plane and lateral incisor is greater with the CBCT compared to the PR (12,32,38). A study showed that the agreement between the methods was 74% in relation to the midline (13). This result provides valuable insights into the diagnostic capabilities of these imaging modalities. This discrepancy in angulation measurements highlights the importance of carefully considering the imaging technique employed, as it can significantly influence the assessment of IC positioning.

In the vertical position of IC, one study found that PR shows a higher position, than CBCT (more apical to the lateral incisor) (13). The other study found more commonly a medium position (middle third of lateral incisor) in both exams (CBCT and PR) (28).

One study compared the effective radiation dose in 10-year-old patients with impacted canines who underwent 2D (PR) and 3D (CBCT) exams, using a thermoluminescent dosimeter system and dosimetric film. The findings showed that the ProMax3D and NewTom5G tomographs resulted in an effective dose of 88 µSv and 170 µSv; while PR resulted in a 4.1 µSv dose (47). This result showed that CBCT generated a higher effective radiation dose when compared with PR. However, 2D scans provided limited IC diagnostic information, due to distortions, superimpositions, and magnification, resulting from the different distances between X-ray source, object, and film (48). These factors can lead to inaccurate and unreliable measurements that can be mitigated by using measurements taken in vertical dimensions, which are more reliable than the horizontal types (49,50). In this study, the mean age of the patients was 17.6 years. CBCT would be a complementary diagnostic tool, in view of the patient’s age. However, this is not a general guideline throughout the entire process of dental development. PR alone is frequently sufficient as a diagnostic tool and CBCT is required only in specific circumstances. CBCT can be requested when resorption of teeth adjacent to the IC is suspected. In such cases, this exam will assist in surgical and orthodontic planning.

There were some limitations to this systematic review. We minimized the bias between the studies included and extracted the utmost homogeneity among them, by using adequate eligibility criteria. Additionally, we selected all studies that evaluated CI by means of PR and CBCT, which used similar methods in children, adolescents, and young adults. The majority of variations among results of the studies occurred due to the various ages of the populations, the number of men and women, sample size, and classification of IC positions. The diversity in the latter classifications, including vertical position (13,28), prevented the authors from including all studies in the meta-analysis.

High heterogeneity was observed in the meta-analysis of angulation to the midline, occlusal plane, and lateral incisor, as well as in the resorption of the lateral incisor and adjacent premolars adjacent to the IC, and in labial and palatal positions. In contrast, the analyses of central incisor resorption and mid-alveolus position indicated low heterogeneity. This variability could be attributed to differences in study populations, methodologies, 2D image quality, and parameters used in CBCT evaluation. Four studies reported the results of resorption according to the number of examiners (8, 26, 11 or 6 examiners) (12,13,27,28).

Seven studies evaluated the position of the IC only on CBCT (9,26,31,33-36). Therefore, they were not included in the meta-analysis. However, they were included in the narrative synthesis. In the other studies that evaluated the labiopalatal position on PR, complementary periapical radiographs, cephalometric radiograph, or study casts were also used (13,25,27). The association of PR with complementary resources could be more reliable than using PR alone to assess the labio-palatal position of the IC. Whereas analysis with the use of CBCT showed the exact position of the IC.

We recommend that further studies use comprehensive and standardized classifications (labial, palatal, mid-alveolus position of IC and mesio-distal position in grades 1 to 4) (41), that report in detail the measurements, thereby allowing comparisons among the results. Furthermore, the authors must justify the sample and report the equipment used (PR and CBCT).

In conclusion, within the limits of the data available for this systematic review, the null hypothesis initially formulated was rejected; CBCT images showed statistically significant differences when compared with PR in the assessment of IC, relative to the mesiodistal and labio-palatal position, angulation to the midline, occlusal plane, lateral incisor, and root resorption of adjacent teeth. CBCT provided clinically relevant information that could contribute to the diagnosis and planning of IC treatment when PR was not sufficient.
